# Healthcare employees’ perspectives on organizational communication about preventive mental health interventions: A focus group study

**DOI:** 10.1371/journal.pone.0334716

**Published:** 2025-10-16

**Authors:** Eline M. Wagelaar, Lisa S. Hogeveen, Nini H. Jonkman, Anne Bakker

**Affiliations:** 1 Department of Occupational Health, OLVG Hospital, Amsterdam, the Netherlands; 2 Department of Family Medicine, Erasmus MC, Rotterdam, the Netherlands; 3 Department of Research and Epidemiology, OLVG Hospital, Amsterdam, the Netherlands; 4 Amsterdam UMC location University of Amsterdam, Psychiatry, Amsterdam Public Health, Amsterdam, the Netherlands; PLoS ONE, UNITED STATES OF AMERICA

## Abstract

The COVID-19 pandemic exposed healthcare employees to stressful situations with possible long-term mental health consequences, stressing the need for supportive interventions. However, in practice healthcare employees’ use of preventive mental health interventions seems limited. Persuasive communication strategies may help to bridge this gap. The aim of this study was to further refine our understanding of healthcare employees’ perspectives on organizational communication about preventive mental health interventions. A qualitative approach was used, consisting of 5 focus groups with hospital workers, either with (K = 3) or without (K = 2) direct patient contact. We used vignettes as a method to discuss three different scenarios to reflect the different levels of prevention (primary, secondary, tertiary). Focus group sessions were audio-taped and transcribed verbatim. Two researchers independently analyzed the data applying thematic analysis within each prevention level. This qualitative study on the employee perspective on communication about preventive mental health interventions demonstrated an overarching funnel movement in which the source and content/channel become increasingly targeted towards the individual employee as mental health symptoms increase. The primary prevention level revealed the theme ‘Multilevel sources of communication and various channels’, the secondary prevention level revealed the theme ‘Specific sources of communication and specific channels’, and the tertiary prevention level revealed the theme ‘A central role for supervisors’. A safe culture in the workplace appeared an important prerequisite for timely discussion of employee mental health. These insights contribute to the development of more tailored organizational communication about mental health of healthcare employees.

## 1. Introduction

Poor mental health in healthcare workers is an emerging problem [1–3]. Importantly, only a part of healthcare employees with mental health problems actually use support, despite the fact that they report a need for it [4–6]. The phenomenon of healthcare employees having difficulty accepting mental health support has previously been described [5,7–9], but COVID-19 has stressed the urgency of this problem [10]. During the pandemic, healthcare employees were exposed to stressful situations [11–15], on top of prior existing high levels of work stress [e.g., 16,17]. This resulted in significant rates of mental health problems such as posttraumatic stress, sleep problems, anxiety, depression, and burnout [18–25]. Although COVID-19 no longer dominates our lives, the pandemic’s side effects, such as inflation, staffing shortages, and supply chain problems, are still pressing challenges for the healthcare industry [26,27]. Post-pandemic research shows that healthcare employees may still suffer from depression symptoms and higher perceived stress levels than the general population [28,29], potentially leading intentions to leave the organization or healthcare sector entirely [30,31].

Preventive mental health interventions both during and beyond pandemic settings may importantly help foster, and if needed help to regain, mental health in healthcare employees [6]. Preventive healthcare can be taught at three chronologically distinct levels [32]. First, interventions to prevent negative mental health consequences that might occur in the first place (primary prevention); next, interventions to prevent progression by identifying and intervening against early indicators of impending ill-health (secondary prevention); and last, when illness occurs, provide early treatment to optimize rapid recovery (tertiary prevention) [6,32]. Interventions at the primary level are preventive in nature in an effort to alleviate the possibility that negative mental health consequences might occur in the first place. Interventions at the secondary level are more ameliorative and seek to intervene early in the disease process for cure or optimal outcomes. And finally, interventions at the tertiary level are aimed at preventing malfunctioning commonly associated with an illness or disease, and supporting people who are experiencing ill effects and require help [32]. In the pandemic context, healthcare leaders were encouraged to take preventive measures in order to support their staff across these three levels [6]. Previous studies have shown that many healthcare organizations indeed offer mental health interventions in the pandemic context, in particular at the primary and secondary prevention level [33–36]. These interventions range from online courses to guide medical staff in dealing with common psychological problems [37–39], self-help interventions by phone [33,34,40] and psychological interventions to release stress [41], such as group activities [33,35].

Despite the variety of interventions being offered, healthcare employees’ need for and use of preventive mental health interventions seems limited, both before and during the pandemic context [e.g., 4,6,17,42]. Denied or dismissed symptoms may result in more long-term adverse outcomes with both health and work-related consequences that are more difficult to treat [28,29,43,44]. According to Billings et al., reasons for not using preventive mental health interventions specifically in the pandemic context relate to additional responsibilities and increased workload, anxiety, uncertainty, and inconsistency of available mental support services [45]. Notably, in the pandemic context these barriers for the use of preventive mental health interventions seem to apply to both front-line medical workers and non-front line medical workers [5]. The prevailing culture of working hard, not complaining, solving your own problems, not showing emotions, and not being sick, within hospitals and among healthcare employees was previously reported to potentially hamper timely use of preventive mental health interventions due to shame, stigma, and mental health illiteracy [7,46–48]. The reluctance to seek preventive mental health interventions in an organizational context is not unique to the hospital setting; and has for instance also been reported in the military context [49,50] and among ambulance personnel [51,52]. In these contexts, virtually the same reasons are given for failure to seek help including being stigmatized, the ability to have time away from work for treatment [53], lack of trust in mental health professionals, anxiety of being perceived as weak or incompetent [54], and the culture within the organization [51].

To overcome some of these barriers and increase the uptake of preventive mental health interventions, important facets of persuasive communication strategies may be utilized [49,55]. According to McGuire’s information processing model, the communication phase is the first and crucial phase of processing persuasive messages in order to eventually modify and maintain new behavior [56]. The communication phase relates to attention, understanding and acceptance [55, p. 57]. Variations in the source of the message, the content of the message, the channel and receiver variables play a role in processing and accepting persuasive messages [57]. A meta-analysis including studies from various workplace settings pointed out that adequate communication can help facilitate the uptake of workplace interventions [48]. Better understanding each of these communication factors within the healthcare setting will provide useful information for designing persuasive messages for healthcare employees. However, so far, a thorough understanding of healthcare employees’ perspective on the communication phase and how messages could be most effectively conveyed to enable behavior modification is unknown.

In short, mental health problems are a potential risk for healthcare employees and the uptake of available preventive interventions seems limited. Persuasive communication strategies could play a role in convincing employees to timely seek help to prevent mental health problems, but a thorough understanding of the employee perspective on organizational communication on this topic within a healthcare setting is lacking. The aim of this study is therefore to further refine our understanding of healthcare employees’ perspectives on organizational communication about preventive mental health interventions.

## 2. Methods

### 2.1. Study design

This study was designed as a phenomenological qualitative study using focus groups with hospital-based healthcare employees. Focus group sessions are an appropriate method to evaluate attitudes, knowledge, and experiences in the healthcare field [58]. We used vignettes as a method to discuss themes. Vignettes are stories about individuals, situations and structures that can refer to important points in the study of perceptions, beliefs, and attitudes [59]. Vignettes are proposed as a helpful and appropriate method if researchers aim to explore the situational context and elucidate influential variables of how actions and occurrences are interpreted and to discuss sensitive experiences in comparison with the ‘normality’ of the vignette [60]. By working with vignettes, we created a safe atmosphere and participants were not forced to reveal whether the examples mentioned came from their own experiences or from behavior of colleagues [61]. The vignettes in our study reflected all three prevention levels (primary, secondary and tertiary prevention) and functioned as framework to classify the vignettes used in the focus groups [62]. The local institutional review board of OLVG (Onze Lieve Vrouwe Gasthuis) Amsterdam approved our study (WO 21.191). We followed the COREQ guideline for the reporting of this study [63]. We pre-registered the study protocol at OSF (OSF.IO/M6GF8). The study was executed conform the preregistered protocol.

### 2.2. Study population and procedures

Employees were recruited through advertisement on the internal website, by email and via posters in OLVG, a large teaching hospital in the Netherlands (> 6000 employees). Moreover, supervisors were asked via email to actively promote study participation among their employees. The recruitment period for this study started on the 4^th^ of April 2022, and ended the 28^th^ of July 2022. Participants were included if they were eighteen years or older and Dutch speaking. A purposive sampling strategy was used to increase the external validity of our results. Based on previous research, we considered the following characteristics as relevant for this variety: gender, age, job profession, number of years of work experience, and need for and use of mental health support [4]. As we were interested in the diversity of participant experiences and opinions, focus groups were mixed, meaning that physicians, nurses, and other healthcare employees participated in the same focus groups [64].

Interested employees received an information letter explaining the aims of the study. Prior to the start of the focus group session, participants signed a written informed consent form. During recruitment, only female employees applied to participate. We subsequently specifically asked male employees, however, without success. As we expected that employees with and without direct patient contact (e.g., administrative, education or human resources staff) could have different perspectives on the study topic especially regarding the channels used, we composed three focus groups consisting solely of participants who directly work with patients, and two focus groups consisting of participants without direct patient contact.

### 2.3. Data collection

The focus group sessions were held between April 2022 and July 2022. Each focus group session was moderated by an independent moderator with communication-background (EW), who encouraged the participants to openly state their viewpoints about the topics. The non-participating group observer (LH or MdH) assisted and made audio recordings of the sessions. There was no prior relationship between the researchers and participants. The moderator used a topic guide that was based on literature and was tested for appropriateness and usefulness in a pilot focus group. In this pilot focus group, consisting of two colleagues, one of whom had a background in health and psychology, and the other in nursing, we discussed the scenarios in the following order: scenario *green*: primary prevention, scenario *orange*: secondary prevention, scenario *red*: tertiary prevention. However, it became clear that discussing the scenarios in reverse order was more useful. So we changed this accordingly. Appendix S1 File shows the discussion guide including introductory elements of the focus groups, the opening question, core questions and concluding elements. The moderator opened each focus group session with an introduction and an open question: What comes to mind when you think about the various forms of communication your hospital provides about mental health support? Subsequently, the major study topic was discussed by means of the three vignettes. Appendix S2 File shows the vignettes we used, presenting a mock-up employee that respectively experiences no, little/emerging, and worsening mental health symptoms reflecting the three different prevention levels. After each session, the moderator and observer exchanged their first impressions of the discussions. Within a few days, the moderator emailed a summary of the session to the participants to evaluate the contribution of each of the participants and to establish whether participants agreed with the summary (member checking). Participants were offered financial compensation for travel expenses and investment of time (€25 voucher per person).

### 2.4. Data analysis

The moderator (EW) and an assistant (SvB) transcribed the audio-recordings of the focus group discussions verbatim. Transcripts of the focus group sessions were imported into ATLAS.ti, a software program for analyzing qualitative data (version 9.1). We used the three prevention levels as framework for the data analysis. We performed a thematic analysis within each level. The thematic analysis was conducted according to the six principles of Braun and Clarke: (1) familiarizing with the data, (2) generating initial codes, (3) searching for themes, (4) reviewing themes, (5) defining and naming themes, and (6) producing the report [65].

The analysis of the focus group discussions proceeded iteratively. Two researchers (EW and LH) analyzed the data. Relevant and important elements were coded with a focus on communication about preventive mental health interventions. These codes were compared and discussed several times in meetings and upon consensus the agreed additional codes were applied to the transcripts. Weekly reflective moments were organized. In case of disagreement, the opinion of a third and fourth researcher was sought (AB and NJ). AB has a background in psycho trauma and NJ in epidemiology and methodology. The findings in the analysis of the first focus group session resulted in a list of important communication elements. We used the findings of the analysis of this focus group session as a guide for the other focus group sessions. After the joint analysis of the first focus group, one researcher (EW) analyzed focus group discussion two, three and five, and another researcher (LH) analyzed focus group discussion four. They coded the relevant text fragments and selected the most important communication elements. These fragments and elements were compared across focus groups and discussed several times in consensus meetings before the final codes were applied to the transcripts. Codes referring to the same phenomenon were grouped into categories, and categories were grouped into themes that represent important and relevant aspects of communication about preventive mental health interventions. This process was repeated several times. After the fifth focus group, data saturation was reached. The whole process was regularly reflected on and twice discussed by the entire research team (EW, LH, AB, NJ). Quotes that underline the main results were presented and were translated from Dutch to English by a near-native English speaker.

## 3. Results

Three of the five focus groups were held with participants who had direct patient contact. Their roles in the hospital consist of: front desk staff, medical social work, nurse and doctor. The other two focus groups consisted of participants without direct patient contact. Function groups they are part of are: HR, education, quality officer, administrative staff and research. All participants were female, with ages between 31 and 64 years old (see Table 1). The focus groups had an average duration of 81 minutes (min. 74 minutes, max 97 minutes).

**Table 1:** Participant characteristics (*n* = 21).

Focus group #	Number (*n*)	Mean age (min.–max.)	Gender (*% female*)	Direct patient contact
Focus group 1	4	56 (35–64)	100%	Yes
Focus group 2	4	36 (33–38)	100%	Yes
Focus group 3	5	52 (34–63)	100%	No
Focus group 4	5	45 (31–58)	100%	Yes
Focus group 5	3	58 (44–63)	100%	No

**Fig 1:**
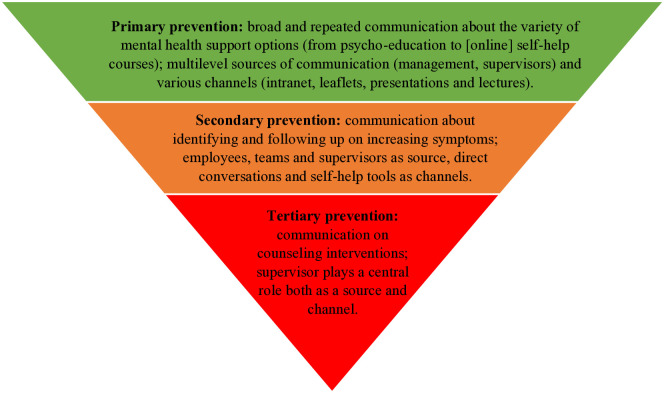
Diagram visualizing healthcare employees’ perspectives on source and channel of preventive mental health communication in an organization.

Across the three different prevention levels, the source and channels narrow down per level and hence show a funnel movement as visualized in Fig 1. Qualitative synthesis on the employee perspective on communication about preventive mental health interventions demonstrated three overarching themes: (1) multilevel sources of communication and various channels for primary prevention, (2) specific sources of communication and specific channels for secondary prevention, and (3) a central role for supervisors for tertiary prevention. These main themes will be discussed in the following sections depicting each of the levels of preventive interventions.

### 3.1. Multilevel sources of communication and various channels for primary prevention

According to participants, communication should be organized at all layers of the organization, stem from different sources, and all organization channels should be used in order to reach employees. Regarding the highest organization layer, supervisors should demonstrate their appreciation for their employees, by means of paying informal visits to the teams, tokens of appreciation, or financial resources for team leisure activities. Informal visits from highly-ranked supervisors for instance during coffee breaks are particularly appreciated, as this would give employees the recognition that their wellbeing is important and really cared about.

*“It’s also very important that, erm, that you really… that the management or maybe even the Board of Directors shows themselves more on the work floor and expresses appreciation. And not just occasionally sending a card, but actually showing up or going for a coffee somewhere.”* (P21)

Board managers could also inform employees with updates via e-mail including messages on mental health.

It was discussed that, for primary prevention, supervisors should particularly have ongoing informal check-in conversations with their employees about their wellbeing, work engagement and work functioning. Supervisors could suggest and facilitate appropriate and available preventive support programs at a regular basis. Daily evaluations or weekly team meetings could help supervisors and their teams to regularly discuss these topics in an informal and psychosocial safe setting.

*“What I also found nice is that the team leaders sometimes said on Friday afternoons, ‘Come on guys, let’s have a drink together.’ At first, I thought, ‘I don’t really feel like it, I want to go home’. But I thought I would sit down anyway, and then you end up having a conversation. And then you can share your story for, well, at least for a little while.”* (P1)

Participants also indicated that employees have a personal responsibility for maintaining their mental health. Communication could facilitate this with specific activities such as taking a walk, exercising, taking breaks, mindfulness, yoga, and stimulate employees to actually apply this.

*“He feels good but is busy, so I think it’s also good to take your breaks. Exercising can help to relieve stress, so I thought it would be good to encourage it, especially for those working from home, to also exercise during work or in-between their duties.”* (P20)

Aside from face-to-face conversations with the board of directors and supervisors, other channels were suggested to reach the population, including monthly lectures by trained colleagues or peer supporters on mental health topics, such as burn-out, depression, aggression, and easy-accessible and retrievable information on the organization’s intranet on mental health interventions such as available mental health programs, testimonials of employees, and an online self-screening instrument.

Some participants stated that an explanation of the ‘why’ of using preventive mental health interventions in this primary prevention level is important in the communication. In this level people feel good, so the usefulness of using preventive interventions should be clearly conveyed, for instance by means of testimonials of colleagues.

### 3.2. Specific sources of communication and specific channels for secondary prevention

At this level, participants believe that communication about preventive mental health interventions should narrow down to the immediate work environment of employees. Different from the primary prevention level, the sources now represent specific stakeholders in the organization, and the channels concentrate on specific forms of communication. Growing awareness among employees for their own mental health is observed, and participants assign increased responsibility for supervisors in terms of initiating conversations about preventive mental health interventions and stimulate the use of available support programs.

At this level it is important that employees recognize and acknowledge their own (worsening) mental health symptoms and take responsibility for any necessary actions. For example, an employee should look for ways of support and initiate a conversation with the supervisor or with the human resource department.

*“He doesn’t recognize it, so what is it due to? So then you would... I think you also have some personal responsibility for yourself, of course.”* (P6)

Participants agreed that supervisors should as well initiate conversations if they signal changed employee functioning or behavior. Supervisors could discuss the relationship between mental health and work functioning and ask the employee what he or she (temporarily or structurally) needs to recover, in terms of for instance reduced workload or other adaptations. If needed, employees from the human resource department may also assist in this.

*“I find it very powerful when someone sets their boundaries and thinks in terms of solutions, right? So it takes two to tango, meaning you have a manager who invites you, and at the same time you also work on your problem-solving ability in terms of what you need from your manager to prevent these kinds of issues.”* (P12)

Results showed that colleagues are a final important source of communication at the secondary prevention level. Participants mentioned that colleagues may look after each other and start a genuine conversation on a colleague’s wellbeing.

*“Colleagues will then still ask, ‘Are you really doing okay?’ and genuinely sit down to listen, not just in passing.”* (P5)

The organization’s intranet could be an important channel to convey messages on mental health, from promoting the availability of workshops or trainings, to providing a self-screening questionnaire with tailored advice based on the outcomes, and testimonials in which colleagues share their experiences.

*“I would create more of a story on the intranet about someone who is close to burnout, to let others know that they’re not the only one, and that’s why OLVG offers mindfulness or yoga.”* (P4)

*“In terms of communication, erm, you could also place a kind of self-test on the intranet in the form of a questionnaire, for instance. Plus with advice, depending on the outcome.”* (P21)

Similar to the primary prevention level, other channels that were mentioned as helpful are hardcopy posters at central places in the building, an explanatory email with the organization’s available preventive mental health interventions, and live interactional communication through clinical lessons, peer-to-peer moments, or workshops.

### 3.3. A central role for supervisors for tertiary prevention

At the tertiary prevention level, participants recognize the supervisor as the most valuable source of communication for employees. The supervisor should follow a structured approach of signaling, discussing, supporting, and monitoring mental health problems. With regard to signaling, a supervisor should be able to observe alarming changes in employee behavior or functioning.

*“I believe that a team leader should know… that’s what I think, right… who your people are, how they present themselves on the work floor. You should notice that something is wrong when something is wrong. You should actually be able to see that in [someone’s] behavior and work, I think.”* (P6)

Upon signaling certain behavior, the supervisor should carefully and sensitively initiate a conversation with the employee. Participants mentioned that it is highly important that the supervisor initiates this conversation, because the employee may no longer be capable of taking action.

*“I really think it’s up to the manager to ask the question, ‘How are you doing, how are you really doing, how do you feel about your work?’ to, say, go just a bit deeper into that iceberg so that these kinds of things, signals, red flags, might come to the surface instead of simply brushing over them too quickly.”* (P20)

Participants mentioned several managerial communication skills they feel are important. For example, it is important for participants that supervisors are capable to normalize the problems of employees and thus remove the guilt of employees that they might feel. In addition, sincere listening and recognition is highly important. It is furthermore important that supervisors take problems seriously, give personal attention to the employee, and guarantee absolute confidentiality of the conversations.

*“Recognition, permission. Yes, that you actually normalize it for him, [saying] ‘We understand it,’ and, erm, that he doesn’t have to feel burdened, and that this is appropriate.”* (P14)

Subsequently, supervisors should support the employee by providing and stimulating the use of available preventive mental health interventions, discuss potential necessary temporary adaptations of work activities to reduce the workload, and provide guidance to the employee in the process of recovery.

*“So indeed, you just need to structurally [change] something in your work or in how you view things, [asking] ‘Does something need to change there?’ And I think there’s also a role for management in that.”* (P19)

Finally, participants believe that follow-up check-in moments between supervisors and employees are necessary to monitor the employee’s wellbeing and repeatedly reconsider adaptations to work activities.

*“Next, I think it’s also important for the team leader to keep an eye on how it’s going, and maintain contact over a period to support him in that.”* (P8)

A crucial underlying condition according to participants regarding prevention concerns a safe psychosocial culture to disclose any emerging mental health problems.

*“It’s about how safe the person feels to express that, you know. That’s very much about that culture, about that appreciation and recognition. So I think that is actually the underlying issue of everything, because you can do and communicate whatever you want, but if that feeling of safety is not there for people, let’s say, then it won’t really resonate or improve.”* (P8)

Apart from the clear task for supervisors at this stage, participants agree that easy access and anonymously available interventions should be available within an organization, where employees can administer themselves. Participants mention that the hierarchical position of supervisors and their busy schedules in some cases hamper their ability and suitability as gatekeepers towards preventive mental health interventions.

*“On the one hand, it’s much more threatening, a team leader or a colleague who addresses you, than finding something anonymous via the intranet without anyone knowing.”* (P4)

## 4. Discussion

The aim of this study was to further refine our understanding of healthcare employees’ perspective on organizational communication about preventive mental health interventions. Results of the focus groups demonstrated three overarching themes: (1) multilevel sources of communication and various channels for primary prevention, (2) specific sources of communication and specific channels for secondary prevention, and (3) a central role for supervisors for tertiary prevention. These themes show an overarching funnel movement in which the source and content/channel become increasingly targeted towards the individual employee as mental health symptoms increase.

Regarding the source of communication messages, our results demonstrated that supervisors and colleagues play an important role, regardless of the prevention level. Important tasks for supervisors include signaling changing behavior, initiating conversations about mental health with the appropriate communication skills, providing guidance to employees in their road to recovery, and regular evaluating this process. Our results are in line with the work of Greenberg and Tracy who showed that supervisors and trained peers in the healthcare setting should be alert for early signs of distress among employees, and should be able to have psychologically savvy supportive conversations [6]. Furthermore, Reynolds and Lehman also showed that colleagues and supervisors in the military context can play an important role in conveying a message to the person who needs mental health support [50]. They showed that stronger feelings of group cohesion, familiarity with services, trust in management, and knowledge of how to get help affected willingness to seek help or recommend help to others. Further, they described that higher ranked colleagues could serve as role models by disclosing their acceptance and use of mental health interventions [49,50]. Importantly, by sharing these experiences publicly in the organization, respected others show they approve the use of preventive mental health interventions and that it did not affect their position within the organization. From ambulance studies, it is also known that the supervisor’s psychosocial safety climate and behavior accounted for some of the variance in levels of common mental health symptoms and well-being [52]. Finally, our results are supported by quantitative work demonstrating the key role of supervisor support with regard to health and wellbeing at work [66].

Regarding the content and channels, our results suggest that at the primary and secondary prevention level, a broad variety of content and channels would be necessary to convey messages on mental health. That is, an organization’s intranet, posters throughout the building, e-mails from the board and live lectures could all be appropriate channels [57] and should be used simultaneously to encourage employees to use preventive measures and to increase more awareness about the topic. These findings are in line with a Dutch national guideline for supporting healthcare employees in preventing mental health problems [17]. However, our results add more concrete tools of communicative interventions per prevention level, that could lead to convincing employees to seek mental support.

Interestingly, from the healthcare employee’s perspective, it is the suggestion that as mental health problems got worse, communication strategies on preventive mental health interventions should concentrate increasingly to the direct work environment. Our data suggest that from an employee perspective, less emphasis is put on employees’ own responsibility to recognize symptoms and seek help. Instead, colleagues and supervisors become increasingly important as symptoms progress. There is a general level of mental health illiteracy in public society resulting in failure to identify own symptoms which may negatively impact help-seeking behavior [67–69]. Difficulties in recognizing symptoms and the need for help may increase as symptoms start to increase [70].

To our knowledge, our study is the first to demonstrate that, based on healthcare employee’s perspectives, supervisor support becomes even more important as mental health complaints in employees start to increase. Ideally, supervisors promote a work environment that stimulates autonomy and self-direction in employees to take responsibility for their own mental health [71,72]. However, our results suggest that employees not only see a role for supervisors in promoting this environment, but also to step in and take a more active role of supporting an employee as mental health complaints get worse. Previous research has shown that supervisors may benefit from training to better understand and support the mental health needs of employees in terms of knowledge, attitudes and self-reported behavior [e.g., 73]. However, effectiveness of such trainings on mental health in employees still have to be proven [74]. Participants of the current study also suggested that colleagues may play a role in signaling emerging mental health problems. Established peer-to-peer support programs in hospital settings [75] or buddy programs [76] in the military context may serve as valuable models.

Our study shows that a safe workplace culture is a prerequisite for discussing and seeking preventive mental health interventions. However, a culture of stigma, shame, and poor mental health literacy were previously reported as common barriers to disclosure and help-seeking [7,46,47,51]. Improving workplace psychosocial safety can be a complex and enduring process, but a growing number of studies demonstrate promising results for behaviorally-based and multifaceted interventions [77,78].

### 4.1. Practice implications

This study provides practical implications for how and by whom to communicate at different prevention levels towards hospital employees. At the primary prevention level, communication should come from different sources, such as supervisors, testimonials of colleagues, and board members, and via different channels, such as the organization’s intranet, e-mails, informal conversations and check-in moments with supervisors or board members, clinical lectures, dedicated team meetings, and online self-screening instruments.

At the secondary prevention level, communication about preventive mental health interventions narrows down to the direct work environment. E-mails, intranet, and clinical lectures become less and one-to-one conversations become more important. Both the employee itself and the supervisor can be stimulated to initiate such a conversation. Offline communication in the form of posters at the ward could help communicate the range of available preventive mental health interventions.

At the tertiary prevention level, according to employees the supervisor has a crucial role. This role consists of signaling changes in work functioning and behavior of employees and check-in with the employee to test these observations and concerns. Supervisor communication skills, such as valuing and -if applicable- normalizing problems, sincere listening and exuding trust are pivotal for a good conversation. The supervisor may then support the employee by referring to available preventive mental health interventions, to discuss the potential need for temporary adaptations to work activities, and to follow-up on these actions. Furthermore, guiding the employee in this process and monitoring the process is also part of the supervisor’s role in this prevention level.

To note, our focus groups included both healthcare employees with and without direct patient contact. There were no noteworthy differences between both groups in terms of perspectives on the preferred sources and channels for designing persuasive messages suggesting that all hospital employees may be addressed similarly on mental health.

### 4.2. Strengths and limitations

A strength of this study is the fruitful focus group discussions, with participants generating ideas from each other and endorsing or expanding on the views expressed by others. The employee perspectives may form an important missing bridge between policy and practice and add to more tailored communication strategies.

A limitation of this study is that the participants do not represent the entire population of the hospital staff, specifically in terms of gender. This may have biased our findings, as there is evidence that women working in healthcare settings have more mental health problems than men [79]. Furthermore, women have different coping strategies to deal with mental health problems [80]. They are more likely to use strategies that involve verbal expressions to others or the self, to seek emotional support, ruminate about problems, and use positive self-talk [80–82]. Moreover, men report higher levels of stigma compared to women [83,84].

Furthermore, according to the persuasion theory of McGuire, the *receiver of the message* plays a role in processing a persuasive message [57]. Whether or not employees have used preventive mental health interventions before, could be of influence, according to Albarracín: “people make decisions to seek information largely due to their existing attitudes on a topic. These prior attitudes are powerful forces that guide exposure to messages, favoring messages that support the prior point of view” [85, p. 192–193]. In our study, 13 of 21 participants previously used preventive mental health interventions. It is possible that because of their experiences with mental healthcare, these employees already had certain beliefs surrounding this topic that could have influenced their perspective on it.

Future research on healthcare employee’s perspectives on organizational communication about preventive mental health interventions may include more diverse samples, including the participation of male employees. This could lead to more representative findings for the entire population of healthcare employees. It should furthermore be noted that our findings reflect the perspective of employees in a Dutch hospital setting, where preventive mental health interventions are readily available. Future studies are needed to investigate perspectives from employees in less high-resource settings as well to determine to what extent employee perspectives on this topic may overlap or differ [86]. Moreover, more insight into the perspectives of supervisors on communication strategies is needed as prior work has shown that perspectives of employees and supervisors on mental health leadership for instance do not necessarily resemble [66,87]. Finally, research is warranted to investigate the impact of improved communication strategies on (intentions for) help-seeking behavior and preventive mental health interventions in terms of uptake during and beyond stressful times. Potential mechanisms that could importantly influence this relationship include attention, comprehension and acceptance of the communication messages within the target group [57].

### 4.3. Conclusion

This qualitative study on the employee perspective on communication about preventive mental health interventions demonstrated three overarching themes: (1) multilevel sources of communication and various channels for primary prevention, (2) specific sources of communication and specific channels for secondary prevention, and (3) a central role for supervisors for tertiary prevention. This shows that the source and channels narrow down per prevention level and hence show a funnel movement. These insights contribute to the development of more tailored organizational communication about mental health of healthcare employees.

## Supporting information

S1 File Discussion guide focus group. (DOCX)

S2 File Vignettes. (DOCX)
