# Supplementary material for: Healthcare employees’ perspectives on organizational communication about preventive mental health interventions: A focus group study
Source: PLoS One. 2025 Oct 16;20(10):e0334716. doi: 10.1371/journal.pone.0334716 (PMC12530549; doi:10.1371/journal.pone.0334716)
Supplement: S1 File — (DOCX) [file pone.0334716.s001.docx]

**S1 Appendix. Discussion guide focus group
Welcome + introduction**

- Welcome
- Thanking the participants for participating
- Introducing the moderator and her role
- Introducing the observant and her role
- Soon: introduction round participants

**Introduction of the research and the focus group**

- Brief introduction of the research
- Brief explanation of what a focus group is
- Aim of the focus groups: gaining insight into the experiences and opinions of participants regarding communication about mental support, so that the way of communicating about mental support can be adjusted where necessary.

**Practical procedure**

- The focus group lasts about 90 minutes. There is a short break after about 45 minutes
- Toilets
- We will produce anonymous transcripts of the focus groups; therefore, we need to audio-record the focus group.

**Rules**

- Don’t talk through each other
- Listen with respect to each other
- Treat the information with strict confidentiality
- Participants don’t necessarily have to agree with each other
- Everything that is said is ok; it is not about “correct” or “wrong” answers

**Introduction round participants**

- Mention your name, position and the department where you work

**Questions?**

- We start with introductory question, then using vignettes to discuss situations about communication and mental support.
- Questions from participants regarding the procedures?
- Does everyone agree that we should start the recording now?

**Opening question**

- What comes to mind when you think about the various forms of communication your hospital provides about mental health support?
- Channel, source, tone of voice, frequency etc.?

STATEMENT: *“OLVG emails a tip on a daily basis, about mental support/preventing mental problems”*

**Introduction core questions**

For the research, it is important to know how the OLVG can prevent possible mental problems. This means that communication from OLVG should be convincing enough that employees take action in time. When it comes to persuasion of a message, we use McGuire's model for this study. This model assumes that someone goes through 3 phases when becoming convinced of a message, namely: **attention**, i.e. how would a message attract attention, **comprehension**, i.e. how would a message be understood, and **acceptance**, i.e. what would make that person accept the message.

We will now look at 3 vignettes and we will ask questions based on them

**Core questions**

**Scenario *red*: tertiary prevention**

Gijs is feeling pretty down. Lately, Gijs has been feeling gloomy. Working in corona time starts to break Gijs down. It's hard, Gijs doesn’t want to budge on it. Gijs feels guilty toward colleagues. Fortunately, Gijs is able to talk well with his partner at home about everything he experiences at work, but he still notices that the situation is not improving. Gijs thinks it would be a good idea to take action in time.

The first vignette involves primary prevention, in other words, “there is nothing going on and Gijs wants to keep it that way”.

What kind of messages/information/communication from OLVG:

- Would get Gijs’ attention?
- Would Gijs understand?
- Would Gijs accept

Write down your answer on a post-it.

**Scenario *orange*: secondary prevention**

Gijs always enjoyed going to work. Lately, however, Gijs has a lot of worries and does not feel himself. Gijs does not recognize this. Nevertheless, Gijs continues to go to work every day. Gijs tries to ignore the feelings, because Gijs has to take care of his patients. They are the most important.

The second vignette involves secondary prevention, in other words, “Gijs has incipient symptoms but it must be prevented from worsening”.

What kind of messages/information/communication from OLVG:

- Would get Gijs’ attention?
- Would Gijs understand?
- Would Gijs accept

Write down your answer on a post-it.

**Scenario *green*: primary prevention**

Gijs is feeling good. Gijs works as a nurse and has been busy the past few months, but actually feels extra useful and valuable to the patients. It gives Gijs a lot of energy. Although Gijs is doing very well, the corona situation remains uncertain. With that, it also remains uncertain how Gijs will experience the coming time. Various mental health activities are offered at work.

The third vignette involves tertiary prevention, or in other words, “the chronicity of Gijs' symptoms must be prevented”.

What kind of messages/information/communication from OLVG:

- Would get Gijs’ attention?
- Would Gijs understand?
- Would Gijs accept

Write down your answer on a post-it.

**Conclusion**

- Summarizing together: what struck you most as a result of this conversation?
- Is there something you want to add to this conversation that could be of importance for the research?
- Thank you for your participation and time.
- Follow-up: we make a summary of the most important findings, and we will email those to you as a check to confirm/adjust/add.
- Handing out declaration form, exit ticket, gift.
