# Supplementary material for: Healthcare employees’ perspectives on organizational communication about preventive mental health interventions: A focus group study
Source: PLoS One. 2025 Oct 16;20(10):e0334716. doi: 10.1371/journal.pone.0334716 (PMC12530549; doi:10.1371/journal.pone.0334716)
Supplement: S2 File — (DOCX) [file pone.0334716.s002.docx]

**S2 Appendix. Vignettes (author’s translation from Dutch into English)**

Vignettes used in the focus groups with participants with direct patient contact:

**Scenario *red*: tertiary prevention**

Gijs is feeling pretty down. Lately, Gijs has been feeling gloomy. Working in corona time starts to break Gijs down. It's hard, Gijs doesn’t want to budge on it. Gijs feels guilty toward colleagues. Fortunately, Gijs is able to talk well with his partner at home about everything he experiences at work, but he still notices that the situation is not improving. Gijs thinks it would be a good idea to take action in time.

**Scenario *orange*: secondary prevention**

Gijs always enjoyed going to work. Lately, however, Gijs has a lot of worries and does not feel himself. Gijs does not recognize this. Nevertheless, Gijs continues to go to work every day. Gijs tries to ignore the feelings, because Gijs has to take care of his patients. They are the most important.

**Scenario *green*: primary prevention**

Gijs is feeling good. Gijs works as a nurse and has been busy the past few months, but actually feels extra useful and valuable to the patients. It gives Gijs a lot of energy. Although Gijs is doing very well, the corona situation remains uncertain. With that, it also remains uncertain how Gijs will experience the coming time. Various mental health activities are offered at work.

Vignettes used in the focus groups with participants with no direct patient contact:

**Scenario *red*: tertiary prevention**

Gijs is feeling pretty down. Lately, Gijs has been feeling gloomy. Working in corona time starts to break Gijs down. It's hard, Gijs doesn’t want to budge on it. Gijs feels guilty toward colleagues. Fortunately, Gijs is able to talk well with his partner at home about everything he experiences at work, but he still notices that the situation is not improving. Gijs thinks it would be a good idea to take action in time.

**Scenario *orange*: secondary prevention**

Gijs always enjoyed going to work. Lately, however, Gijs has a lot of worries and does not feel himself. Gijs does not recognize this. Nevertheless, Gijs continues to go to work every day. Gijs tries to ignore the feelings, because Gijs has to meet his deadline.

**Scenario *green*: primary prevention**

Gijs is feeling good. Gijs has been busy the past few months, but actually feels extra useful and valuable. It gives Gijs a lot of energy. Although Gijs is doing very well, the corona situation remains uncertain. With that, it also remains uncertain how Gijs will experience the coming time. Various mental health activities are offered at work.
